# Resistive phase transition of the superconducting Si(111)-($\sqrt{7}\times \sqrt{3}$)-In surface

**DOI:** 10.1186/1556-276X-8-167

**Published:** 2013-04-11

**Authors:** Takashi Uchihashi, Puneet Mishra, Tomonobu Nakayama

**Affiliations:** 1 International Center for Materials Nanoarchitectonics (MANA), National Institute for Materials Science (NIMS), , 1–1, Namiki, Tsukuba, Ibaraki, 305–0044, Japan

**Keywords:** Surface reconstruction, Silicon, Indium, Superconductivity, Electron transport, Fluctuation effects, Vortex flow

## Abstract

Recently, superconductivity was found on semiconductor surface reconstructions induced by metal adatoms, promising a new field of research where superconductors can be studied from the atomic level. Here we measure the electron transport properties of the Si(111)-($\sqrt{7}\times \sqrt{3}$)-In surface near the resistive phase transition and analyze the data in terms of theories of two-dimensional (2D) superconductors. In the normal state, the sheet resistances (2D resistivities) *R*_□_ of the samples decrease significantly between 20 and 5 K, suggesting the importance of the electron-electron scattering in electron transport phenomena. The decrease in *R*_□_ is progressively accelerated just above the transition temperature (*T*_*c*_) due to the direct (Aslamazov-Larkin term) and the indirect (Maki-Thompson term) superconducting fluctuation effects. A minute but finite resistance tail is found below *T*_*c*_ down to the lowest temperature of 1.8 K, which may be ascribed to a dissipation due to free vortex flow. The present study lays the ground for a future research aiming to find new superconductors in this class of materials.

## Background

Semiconductor surface reconstructions induced by metal adatoms constitute a class of two-dimensional (2D) materials with an immense variety [1,2]. They are considered one form of atomic layer materials which can possess novel electronic properties and device applications [3,4]. Recently, superconductivity was measured by scanning tunneling microscopy (STM) for atomically thin Pb films [5,6] and three kinds of Si(111) surface reconstructions: SIC-Pb, ($\sqrt{7}\times \sqrt{3}$)-Pb, and ($\sqrt{7}\times \sqrt{3}$)-In [7]. This discovery was followed by a demonstration of macroscopic superconducting currents on Si(111)-($\sqrt{7}\times \sqrt{3}$)-In by direct electron transport measurements [8]. These findings are important because they enable us to create superconductors from the atomic level using state-of-the-art nanotechnology. In addition, the space inversion symmetry breaking due to the presence of surface naturally leads to the Rashba spin splitting [9,10] and may consequently help realize exotic superconductors [11].

In reference[8], we have unambiguously clarified the presence of Si(111)-($\sqrt{7}\times \sqrt{3}$)-In (referred to as ($\sqrt{7}\times \sqrt{3}$)-In here) superconductivity. However, systematic analysis on electron transport properties above and below the transition temperature (*T*_*c*_) is still lacking. For example, 2D superconductors are known to exhibit the precursor of phase transition due to the thermal fluctuation effects just above *T*_*c*_[12-14]. Superconductivity is established below *T*_*c*_, but vortices can be thermally excited in a 2D system. Their possible motions can cause the phase fluctuation and limit the ideal superconducting property of perfect zero resistance [15]. These fundamental properties should be revealed before one proceeds to search for new superconductors in this class of 2D materials.

In this paper, the resistive phase transition of the ($\sqrt{7}\times \sqrt{3}$)-In surface is studied in detail for a series of samples. In the normal state, the sheet resistances (2D resistivities) *R*_□_ of the samples decrease significantly between 20 and 5 K, which amounts to 5% to 15% of the residual resistivity *R*_n,res_. Their characteristic temperature dependence suggests the importance of electron-electron scattering in electron transport phenomena, which are generally marginal for conventional metal thin films. *T*_*c*_ is determined to be 2.64 to 2.99 K and is found to poorly correlate with *R*_n,res_. The decrease in *R*_□_ is progressively accelerated just above *T*_*c*_ due to the superconducting fluctuation effects. Quantitative analysis indicates the parallel contributions of fluctuating Cooper pairs due to the direct (Aslamazov-Larkin term) and the indirect (Maki-Thompson term) effects. A minute but finite resistance tail is found below *T*_*c*_ down to the lowest temperature of 1.8 K, which may be ascribed to a dissipation due to free vortex flow.

## Methods

**Figure 1:**
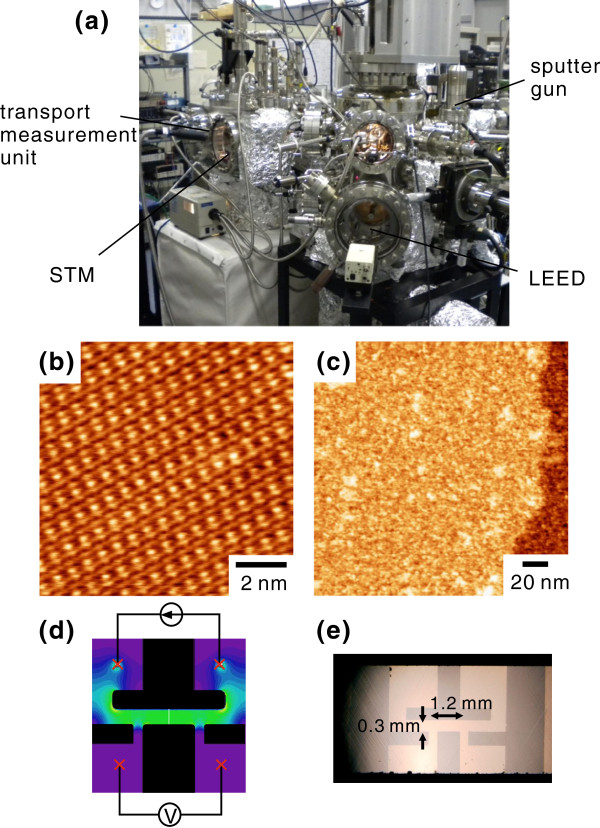
**Instrumentation and sample preparation.** The whole procedure from the sample preparation through the transport measurement was performed in a home-built UHV apparatus without breaking vacuum (**a**). Typical STM images of a ($\sqrt{7}\times \sqrt{3}$)-In sample before (**b**) (*V*_sample_ = −0.015 V) and after (**c**) (*V*_sample_=2.0 V) are displayed. (**d**) The design of sample patterning in the black area shows the Ar ^+^-sputtered region. The color indicates the degree of calculated current density (green, high; purple, low). (**e**) Optical microscope image of a patterned sample.

The experimental method basically follows the procedure described in reference [8] but includes some modifications. The whole procedure from the sample preparation through the transport measurement was performed in a home-built ultrahigh vacuum (UHV) apparatus without breaking vacuum (see Figure 1a) [16,17]. First, the ($\sqrt{7}\times \sqrt{3}$)-In surface was prepared by thermal evaporation of In onto a clean Si(111) substrate, followed by annealing at around 300°C for approximately 10 s in UHV [18-20], and was subsequently confirmed by low-energy electron diffraction and STM. The sample was then patterned by Ar ^+^ sputtering through a shadow mask to define the current path for four-terminal resistance measurements. Typical STM images before and after sputtering are displayed in Figure 1b,c, respectively. The former shows a clear periodic structure corresponding to the $\sqrt{7}\times \sqrt{3}$ unit cell, while the latter shows a disordered bare silicon surface.

We note that, although the nominal coverage of the evaporated In is more than several monolayers (ML), post annealing removes surplus In layers and establishes the ($\sqrt{7}\times \sqrt{3}$)-In surface. The In coverage of this surface reconstruction was originally proposed to be 1 ML for the ‘hexagonal’ phase (($\sqrt{7}\times \sqrt{3}$)-In-hex) and 1.2 ML for the ‘rectangular’ phase (($\sqrt{7}\times \sqrt{3}$)-In-rect) [18], where 1 ML corresponds to the areal density of the top-layer Si atoms of the ideal Si(111) surface. However, recent theoretical studies point to the coverages of 1.2 ML for the ($\sqrt{7}\times \sqrt{3}$)-In-hex and of 2.4 ML for the ($\sqrt{7}\times \sqrt{3}$)-In-rect [21,22]. For our experiments, the dominant phase is likely to be the ($\sqrt{7}\times \sqrt{3}$)-In-hex judging from the resemblance of the obtained STM images (Figure 1b) to the simulated image of the ($\sqrt{7}\times \sqrt{3}$)-In-hex (Figure two, panel b in [22]). The relation between the surface structure and the superconducting properties is intriguing and will be the subject of future work.

In the previous study, van der Pauw’s measurement was adopted to check the anisotropy of electron conduction and to exclude the possibility of spurious supercurrents. In this setup, however, transport characteristics should be analyzed with care because the spatial distribution of bias current is not uniform. To circumvent this problem, in the present study, we adopted a configuration with a linear current path between the voltage terminals (Figure 1d). The black regions represent the area sputtered by Ar ^+^ ions through the shadow mask. The figure also shows the current density distribution calculated by the finite element method in color scale, which confirms that it is homogeneous between the voltage probes. This allows us to determine the sheet resistance *R*_□_ of the sample in a more straightforward way: *R*_□_=(*V*/*I*)×(*W*/*L*), where *V* is the measured voltage, *I* is the bias current, *W*=0.3 mm is the width of the current path, and *L*=1.2 mm is the distance between the voltage probes. Figure 1e shows the optical microscope image of a sample, confirming the clear boundary between the shadow-masked and sputtered regions. Although the sputtering was very light, the resulting atomic-scale surface roughening was enough to make an optical contrast between the two regions.

Following the sample preparation, four Au-coated spring probes were brought into contact with the current/voltage terminal patterns in a UHV-compatible cryostat. Four-terminal zero bias sheet resistance *R*_□_ was measured with a DC bias current *I*=1 µA, and the offset voltage was removed by inverting the bias polarity. To access the electron conduction only through the ($\sqrt{7}\times \sqrt{3}$)-In surface at low temperatures, Si(111) substrates without intentional doping (resistivity *R*>1,000 *Ω* cm) were used. Leak currents through the substrate and the Ar ^+^-sputtered surface region were undetectably small below 20 K, which allowed precise measurements in this temperature region.

## Results and discussion

### Electron transport properties above *T*_*c*_

**Figure 2:**
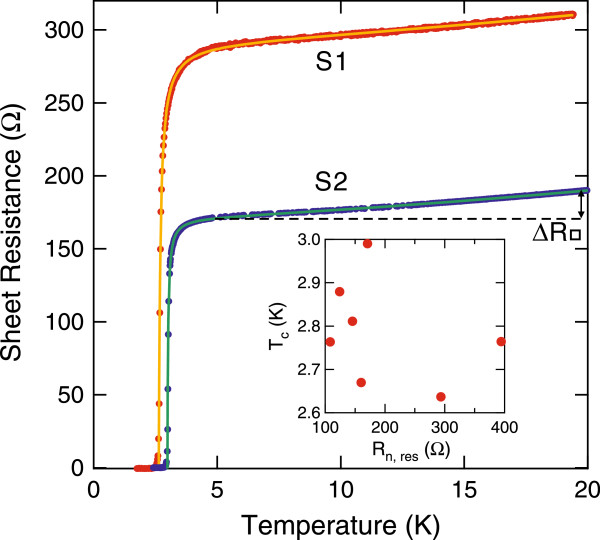
**Electron transport properties above *T***_***c***_**.** The red and blue dots represent the temperature dependences of sheet resistance *R*_□_ for sample S1 and S2, respectively, while the yellow and green lines are the results of fitting analysis using Equations 1 to 3. *ΔR*_□_ is defined as the decrease in *R*_□_ between 20 and 5 K. The inset shows *T*_*c*_ as a function of *R*_n,res_, revealing no clear correlation between them.

In the present study, we investigated seven samples referred to as S1, S2,... and S7. They were prepared through the identical procedure as described above, but due to subtle variations in the condition, they exhibit slightly different electron transport properties. As representative data, the temperature dependences of sheet resistance *R*_□_ for S1 and S2 are displayed in Figure 2 (red dots, S1; blue dots, S2). *R*_□_ drops to zero at *T*_*c*_≈2.6 K for S1 and at *T*_*c*_≈3.0 K for S2, consistent with the previous study on the superconducting phase transition [8]. The rest of the samples show the same qualitative behaviors. As shown below, S1 and S2 exhibit the lowest and the highest *T*_*c*_, respectively, among all the samples. Here we note two distinctive features: (i) For the high-temperature region of 5 K<*T*<20 K, *R*_□_ decreases with decreasing *T*, i.e., *dR*_□_/*dT*>0. The temperature dependence of *R*_□_ is slightly nonlinear with a concave curvature, i.e., *d*^2^*R*_□_/*dT*^2^>0. (ii) The decrease in *R*_□_ is progressively accelerated as *T* approaches *T*_*c*_.

The data were analyzed to deduce characteristic parameters as follows. Feature (i) can be phenomenologically expressed by the 2D normal state conductivity *G*_□,n_ of the following form:

(1) $$G_{□,n}={\left(R_{\text{n,res}}+CT^{a}\right)}^{-1}$$

where *R*_n,res_ is the residual resistance in the normal state, *C* is the prefactor, and *a* is the exponent of the power-law temperature dependence. Feature (ii) is naturally attributed to the superconducting fluctuation effects [14]. Just above *T*_*c*_, parallel conduction due to thermally excited Cooper pairs adds to the normal electron conduction (Aslamazov-Larkin (AL) term), and this effect is enhanced in a 2D systems [12]. The 2D conductivity due to the Cooper pair fluctuation *G*_□,sf_ takes the following form:

(2) $$G_{□,\text{sf}}=\frac{1}{R_{0}}\frac{T}{T-T_{c}}$$

where *R*_0_ is a temperature-independent constant. In addition to this direct effect, another indirect contribution may be important near *T*_*c*_, which originates from the inertia of Cooper pairs after decaying into pairs of quasiparticles (Maki-Thompson (MT) term) [13]. Since its temperature dependence is similar to Equation 2 but involves more material-dependent parameters, we combine these two effects and adopt Equation 2. Importantly, for the pure AL term, $R_{0}=16\hbar /e^{2}=65.8\;\text{k}\Omega$ regardless of the thickness. Then the total sheet resistance above *T*_*c*_ is given by the following equation:

(3) $$R_{□}={\left(G_{□,n}+G_{□,\text{sf}}\right)}^{-1}.$$

The experimental data were fitted excellently using Equations 1 to 3 with *R*_n,res_, *C*, *a*, *R*_0_, and *T*_*c*_ being fitting parameters, as shown in Figure 2 (yellow line, S1; green line, S2). Since Equation 2 is only valid for *T*>*T*_*c*_, the data of the normal state region (defined as *R*_□_>50 *Ω*) were used for the fitting. All parameters thus determined are listed in Table 1 for the seven samples. We note that the obtained values for *R*_0_ are all smaller by a factor of 2.4 to 5.4 than *R*_0_=65.8 k*Ω* for the AL term. This indicates that the observed fluctuation-enhanced conductivities originate from both AL and MT terms. We also tried to fit the data by explicitly including the theoretical form for the MT term [13], but this resulted in poor fitting convergence.

**Table 1:** **Summary of the fitting analysis on the resistive transition of the** ($\sqrt{7}\times \sqrt{3}$)**-In surface**

**Sample**	***R***_**0**_** (k*Ω*)**	***R***_**n,res**_** (*Ω*)**	***T***_***c***_**(K)**	**b**	***ΔR***_**□**_**/*R***_**n,res**_**(%)**
S1	12.1	293	2.64	1.80	8.0
S2	20.0	171	2.99	1.54	10.8
S3	15.6	146	2.81	1.78	12.6
S4	17.6	108	2.76	1.67	15.3
S5	27.7	394	2.76	1.86	5.0
S6	14.3	160	2.67	1.69	11.5
S7	20.9	124	2.88	1.48	13.7

The determined *T*_*c*_ ranges from 2.64 to 2.99 K. This is in reasonable agreement with the previously determined value of *T*_*c*_=2.8 K, but there are noticeable variations among the samples. The normal residual resistance *R*_n,res_ also shows significant variations, ranging from 108 to 394 *Ω*. These two quantities, *T*_*c*_ and *R*_n,res_, could be correlated because a strong impurity electron scattering might cause interference-driven electron localization and suppress *T*_*c*_[23]. However, they are poorly correlated, as shown in the inset of Figure 2. This is ascribed to possible different impurity scattering mechanisms determining *R*_n,res_ and *T*_*c*_ as explained in the following. Electron scattering should be strong at the atomic steps because the surface layer of ($\sqrt{7}\times \sqrt{3}$)-In is severed there. Therefore, they contribute to most of the observed resistance [8,24]. However, the interference between scatterings at the atomic steps can be negligibly weak if the average separation between the atomic steps *d*_av_ is much larger than the phase relaxation length *L*_*ϕ*_. This is likely to be the case because *d*_av_≈400 nm for our samples, and *L*_*ϕ*_ is several tens of nanometer for typical surfaces [25]. In this case, electron localization and resultant suppression of *T*_*c*_ are dominated by other weaker scattering sources within the size of *L*_*ϕ*_, not by the atomic steps that determine *R*_n,res_.

The exponent *a* was determined to be 1.48 to 1.85 in accordance with feature (i). This might be seen as a typical metallic behavior due to the electron-phonon scattering. However, this mechanism would lead to *R*_e-ph_∝*T* for *T*>*Λ*_D_ and *R*_e-ph_∝*T*^5^ for *T*≪*Λ*_D_[26], neither of which is consistent with the observed temperature dependence. (Here *R*_e-ph_ is the resistance due to the electron-phonon scattering, and *Λ*_D_ is the Debye temperature.) Considering the exponent *a* to be slightly smaller than 2, we attribute its origin to the electron-electron scattering. In a 2D Fermi liquid, it leads to a resistivity *R*_e−e_ with the following form [27],

(4) $$R_{e-e}=C^{\prime }T^{2}\ln\frac{\varepsilon_{F}}{k_{B}T}$$

where *C*^′^ is a proportional constant, *ε*_F_ is the Fermi energy, and *k*_B_ is the Boltzmann constant. The log term in Equation 4 results in a weaker temperature dependence than that in a 3D Fermi liquid (∝*T*^2^). Fitting the data with Equation 4 instead of the *CT*^*a*^ term in Equation 1 gives *ε*_F_≈0.1 eV, although the uncertainty is quite large.

We note that a decrease in resistance in a conventional metal film is usually very small in this temperature range. For example, it is less than 1% within the range of 2<*T*<20 K for 2-nm-thick single-crystal Nb films, although *R*_□_=122 *Ω* of the film is comparable to the observed *R*_n,res_ in the present experiment [28]. For a metal thin film with a large resistance, *R*_□_ even increases slightly with decreasing *T* as a consequence of the electron localization [29]. In clear contrast, a decrease in *R*_□_ between 20 and 5 K in our samples, *ΔR*_□_, amounts to as much as 5% to 15% of *R*_n,res_ (see Figure 2 and Table 1). In this sense, the observed temperature dependence is rather unusual. The ($\sqrt{7}\times \sqrt{3}$)-In surface studied here has an atomic-scale dimension in the normal direction and may thus have an enhanced electron-electron interaction because of insufficient electrostatic screening. In comparison, the contribution from the electron-phonon interaction can be smaller because it decreases rapidly at low temperatures as *R*_e-ph_∝*T*^5^.

### Residual resistance in the superconducting phase below *T*_*c*_

**Figure 3:**
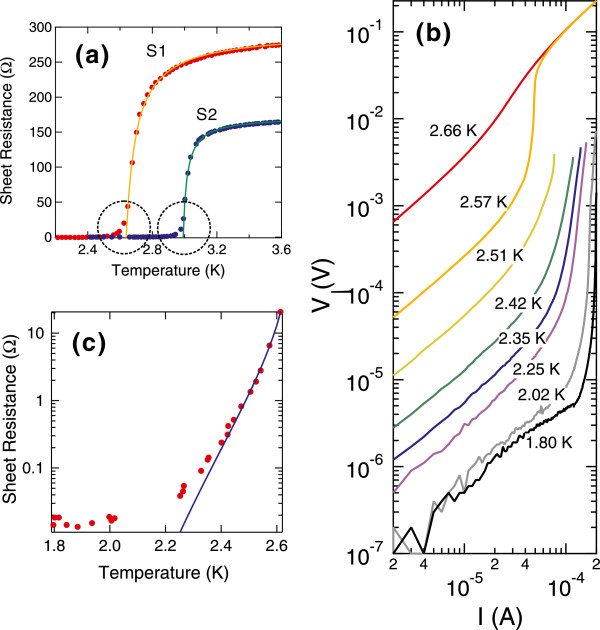
**Residual resistance in the superconducting phase below *T***_***c***_**.** (**a**) Magnified view of Figure 2 around *T*_*c*_. The broken circles indicate the presence of residual resistances below *T*_*c*_. (**b**) Temperature dependence of the *I-V* characteristics of sample S1 below *T*_*c*_. The data are plotted in the log-log scales. The measured temperatures are indicated in the graph. (**c**) Red dots show the sheet resistance determined from the low-bias linear region of the *I-V* characteristics of sample S1. The blue line shows the result of the fitting analysis using Equation 6 within the range of 2.25 K<*T*<2.61 K while *T*_*c*_=2.64 K is fixed.

The superconducting fluctuation theories state that *R*_□_ becomes exactly zero at *T*_*c*_, as indicated by Equation 2. However, a close inspection into the magnified plots (Figure 3a) reveals that *R*_□_ has a finite tail below *T*_*c*_. To examine whether *R*_□_ becomes zero at sufficiently low temperatures, we have taken the current-voltage (*I-V*) characteristics of sample S1 below *T*_*c*_ down to the lowest temperature of 1.8 K. Figure 3b displays the data in the log-log plot form. Although the *I-V* characteristics exhibit strong nonlinearity at the high-bias current region, they show linear relations around the zero bias at all temperatures. The sheet resistances *R*_□_ determined from the linear region of the *I-V* curves are plotted in Figure 3c as red dots. *R*_□_ decreases rapidly as temperature decreases from *T*_*c*_, but it becomes saturated at approximately 2×10^−2^*Ω* below 2 K.

This residual resistance can be attributed to dissipation due to free vortex flow, which is caused by the Lorentz force between the magnetic flux and the current [15], since the stray magnetic field is not shielded in the experiment. The sheet resistance due to the free vortex flow *R*_□,v_ is given by the following equation:

(5) $$R_{□,v}=2\Pi \xi^{2}R_{□,n}B/\Phi_{0}$$

where *ξ* is the Ginzburg-Landau coherence length, *R*_□,n_ is the normal sheet resistance of the sample, *B* is the magnetic field perpendicular to the suface plane, and *Φ*_0_=*h*/2*e* is the fluxoid quantum. A crude estimation using *ξ*=49 nm,*R*_□,n_=290 *Ω*, and *B*=3×10^−5^ T gives *R*_□,v_=6.3×10^−2^*Ω*, which is in the same order of magnitude as the observed value of approximately 2×10^−2^*Ω*. We note that *ξ*=49 nm was adopted from the value for the Si(111)-SI-Pb surface [7], and *ξ* is likely to be smaller here considering the difference in *T*_*c*_ for the two surfaces. The present picture of free vortex flow at the lowest temperature indicates that strong pinning centers are absent in this surface superconductor. This is in clear contrast to the 2D single-crystal Nb film [28], where the zero bias sheet resistance was undetectably small at sufficiently low temperatures. In accordance with it, the presence of strong vortex pinning was concluded from the observation of vortex *creep* in [28]. This can be attributed to likely variations in local thickness of the epitaxial Nb film at the lateral scale of vortex size [30]. The absence of ‘local thickness’ variation in the present surface system may be the origin of the observed free vortex flow phenomenon.

As mentioned above, *R*_□_ rapidly decreases just below *T*_*c*_. This behavior could be explained by the Kosterlitz-Thouless (KT) transition [31,32]. In a relatively high-temperature region close to *T*_*c*_, thermally excited free vortices cause a finite resistance due to their flow motions. As temperature decreases, however, a vortex and an anti-vortex (with opposite flux directions) make a neutral bound-state pair, which does not move by current anymore. According to the theory, all vortices are paired at *T*_*K*_, and resistance becomes strictly zero for an infinitely large 2D system. The temperature dependence of *R*_□_ for *T*_*K*_<*T*<*T*_*c*_ is predicted as follows:

(6) $$R_{□}=C^{\prime \prime }\exp\left\{-2{\left[\frac{b(T_{c}-T)}{T-T_{K}}\right]}^{1/2}\right\}$$

where *C*^′′^ is a prefactor, and *b* a material-dependent constant. For this transition to be observable, the transverse penetration depth *λ*_⊥_ for magnetic field must be larger than the sample size so that vortices can interact with each other logarithmically as a function of the mutual distance. The ultimate atomic-scale thickness of the present system leads to a very large *λ*_⊥_ in the order of millimeters [8], thus making it a candidate for observing the KT transition. We fitted the experimental data of *R*_□_ using Equation 6 within the range of 2.25 K<*T*<2.61 K while *T*_*c*_=2.64 K is fixed. The result is shown in Figure 3c as a blue line. The reasonable fitting over two orders of magnitude in *R*_□_ points to the precursor of the KT transition. The obtained value of *T*_*K*_=1.69 K is deviated from the relation [31]

(7) $$T_{K}/T_{c}\approx {\left(1+0.17R_{□,n}/R_{c}\right)}^{-1}$$

where $R_{c}=\hbar /e^{2}=4.11\;k\Omega$ and *R*_□,n_ are identified with *R*_n,res_=293 *Ω* of sample S1 here. However, Equation 7 is derived from the assumption of the dirty-limit BCS superconductor, which is not applicable to the ($\sqrt{7}\times \sqrt{3}$)-In surface with high crystallinity. Unfortunately, the present experimental setup does not allow us to observe the expected temperature dependence of Equation 6 down to *T*_*K*_ because of the presence of the stray magnetic field. Furthermore, the predicted *I-V* characteristics *V*∝*I*^*a*^ where the exponent *a* jumps from 1 to 3 at *T*_*K*_ should be examined to conclude the observation of the KT transition [31,32]. Construction of a UHV-compatible cryostat with an effective magnetic shield and a lower achievable temperature will be indispensible for such future studies.

## Conclusions

We have studied the resistive phase transition of the ($\sqrt{7}\times \sqrt{3}$)-In surface in detail for a series of samples. In the normal state, the sheet resistances *R*_□_ of the samples decrease significantly between 20 and 5 K, which amounts to 5% to 15% of the residual resistivity *R*_res_. Their characteristic temperature dependence suggests the importance of electron-electron scattering in electron transport phenomena. The poor correlation between the variations in *T*_*c*_ and *R*_res_ indicate different mechanisms for determining these quantities. The decrease in *R*_□_ was progressively accelerated just above *T*_*c*_ due to the superconducting fluctuation effects. Quantitative analysis indicates the parallel contributions of fluctuating Cooper pairs corresponding to the AL and MT terms. A minute but finite resistance tail was found below *T*_*c*_ down to the lowest temperature of 1.8 K, which may be ascribed to a dissipation due to free vortex flow. The interpretation of the data based on the KT transition was proposed, but further experiments with an improved cryostat are required for the conclusion.

## Competing interests

The authors declare that they have no competing interests.

## Authors’ contributions

TU and PM carried out the sample fabrication/characterization and the electron transport measurements. TU and TN conceived of the study. TU analyzed the data and drafted the manuscript. All authors read and approved the final manuscript.
